# Assessment of metals bioavailability to vegetables under field conditions using DGT, single extractions and multivariate statistics

**DOI:** 10.1186/1752-153X-6-119

**Published:** 2012-10-18

**Authors:** Marin Senila, Erika Andrea Levei, Lacrimioara Ramona Senila

**Affiliations:** 1 INCDO-INOE 2000, Research Institute for Analytical Instrumentation, ICIA, 67 Donath, 400293, Cluj-Napoca, Romania

**Keywords:** DGT, Effective concentration, Chemical extraction, Metal, Vegetable, Multivariate statistics

## Abstract

**Background:**

The metals bioavailability in soils is commonly assessed by chemical extractions; however a generally accepted method is not yet established. In this study, the effectiveness of Diffusive Gradients in Thin-films (DGT) technique and single extractions in the assessment of metals bioaccumulation in vegetables, and the influence of soil parameters on phytoavailability were evaluated using multivariate statistics. Soil and plants grown in vegetable gardens from mining-affected rural areas, NW Romania, were collected and analysed.

**Results:**

Pseudo-total metal content of Cu, Zn and Cd in soil ranged between 17.3-146 mg kg^-1^, 141–833 mg kg^-1^ and 0.15-2.05 mg kg^-1^, respectively, showing enriched contents of these elements. High degrees of metals extractability in 1M HCl and even in 1M NH_4_Cl were observed. Despite the relatively high total metal concentrations in soil, those found in vegetables were comparable to values typically reported for agricultural crops, probably due to the low concentrations of metals in soil solution (C_soln_) and low effective concentrations (C_E_), assessed by DGT technique. Among the analysed vegetables, the highest metal concentrations were found in carrots roots. By applying multivariate statistics, it was found that C_E_, C_soln_ and extraction in 1M NH_4_Cl, were better predictors for metals bioavailability than the acid extractions applied in this study. Copper transfer to vegetables was strongly influenced by soil organic carbon (OC) and cation exchange capacity (CEC), while pH had a higher influence on Cd transfer from soil to plants.

**Conclusions:**

The results showed that DGT can be used for general evaluation of the risks associated to soil contamination with Cu, Zn and Cd in field conditions. Although quantitative information on metals transfer from soil to vegetables was not observed.

## Background

The concentrations of toxic metals in soils have continuously increased as a result of anthropogenic activities through inputs mainly from mining, municipal wastes, road traffic or fuel burning. In addition to their toxicity, metals persist in soil for long times and have the capacity to be transferred into the food chain [1,2] thus the assessment of their content in soil and the estimation of their transfer rates to vegetation are of great interest [3]. The soil quality guidelines are usually based on total metal content, although it is generally accepted that total metal content include both bioavailable and non-bioavailable fractions [4]. The estimation of bioavailable metal fractions is, generally, based on single or sequential extraction procedures [5,6], but these provide only a classification of metal fractions in the soil compartments. Additionally, in the pretreatment stage of these procedures, the physical–chemical equilibrium in soil may be affected [7]. The Diffusive Gradients in Thin-films (DGT) is a promising tool in the assessment of bioavailable fraction of metals in soil. It was developed for measuring labile metal species in aqueous systems, [8,9] but its applicability was also extended to sediments and soils. In soils, DGT mimic the uptake of metals by the plant roots because, like these, decrease metal concentrations in their vicinity and responds to metals re-supplied from soil solution and solid phase. Good correlations between available fraction of some metals in soils measured by DGT and their concentration in plants, after pot experiments, were reported [10-12].

Although DGT technique is expected to be superior to the conventional extraction techniques its effectiveness in assessing metals transfer to plants is still under debate. Moreover, there are only a few papers that present data on DGT method use for assessment of metals availability to plants, especially in field trials. However, DGT does not account the processes from rhizosphere, such as root-induced pH changes and exudation of metal complexing compounds that influence metal bioavailability to plants [13-15]. Consequently, to date, an universally accepted procedure for metals bioavailability prediction does not exist and the development and validation of such methods that allows the estimation of the risks posed by the occurrence of toxic metals in soils are necessary [15].

The aim of this study was to assess the metals bioavailability in garden soils from rural areas affected by mining activities using DGT technique and single extractions, to correlate the potentially bioavailable metals fractions with the soil properties and to study the effectiveness of different methods to predict metals uptake in edible parts of several vegetable species. Multivariate statistical approaches were used to reveal the relationships between soil properties, metals concentrations in soil, and their bioaccumulation in vegetables.

## Results and discussion

### *Pseudo*-total metal contents and characteristics of soils

The general characteristics of the soils are presented in Table 1. Soil pH values were in the neutral range (6.7–7.8), while the soil organic carbon (OC) ranged between 1.5-5.4% showing the different fertilization degree of soils. The cation exchange capacity (CEC) values were in the range of 13.1-25.7 meq 100g^-1^ with average value of 19.7 meq 100g^-1^. The pseudo-total content of Cu ranged between 17.3 and 146 mg kg^-1^ dry weight (dw) with the average value below the alert level (100 mg kg^-1^) for soils from sensitive areas established by Romanian legislation (MO 756:1997). Nevertheless, in four sampling points from BM, one sampling point from SA and one from RC this value was exceeded. For Zn, the pseudo-total content was in the range of 141–833 mg kg^-1^ dw, with the average content exceeding the alert level. Additionally, in 20% of the sampling points the intervention level for Zn was exceeded. The content of Cd in all analysed samples were below the alert level, however 33% of soil samples exceeded the worldwide range of Cd in soils (0.06–1.1 mg kg^-1^, mean of 0.53 mg kg^-1^) [16], showing an enrichment of soil Cd content. The obtained results are confirmed by previous studies that reported similar contamination levels with metals of soils from the Baia Mare area [17,18].

**Table 1:** General characteristics of soils collected from the study area

**Sites**		**pH**	**OC (%)**	**CEC (meq 100g**^**-1**^**)**	**Total concentration (mg kg**^**-1**^**dw)**		
					**Cu**	**Zn**	**Cd**
BM	Mean	7.1	3.2	20.0	79.8	550	1.29
	Range	6.7-7.5	1.9-4.8	13.1-25.7	29.1-122	222-833	0.39-2.05
RC	Mean	7.2	2.9	18.6	48.2	393	0.85
	Range	6.8-7.6	1.5-4.0	14.5-22.1	22.8-106	178-660	0.43-1.44
SA	Mean	7.3	4.2	20.6	50.9	394	0.56
	Range	6.9-7.8	3.3-5.4	17.8-24.1	17.3-146	141-491	0.15-1.34
AL*		-	-	-	100	300	3
IL**		-	-	-	200	600	5

* AL-alert level for soil in sensitive (residential and agricultural) areas (Ministerial Order 756:1997).

** IL- intervention level for soil in sensitive (residential and agricultural) areas.

### Chemical single extraction procedures for metals bioavailability prediction

Table 2 shows the ranges and the averages of extractable heavy metal contents obtained by 1M HCl and 1M NH_4_Cl extractions. Compared to the pseudo-total metal contents, the average percentage of the HCl extracted metal fractions were relatively high (64% Cu, 32% Zn and 75% Cd). It is recognized that 1M HCl not attack the silicates and thus the amounts of metals measured by this extraction procedure estimates better the bioavailable fraction than the total metals content [19]. Significant positive relationship between pseudo-total metal and 1M HCl extractable metal contents were obtained by linear regression, with regression coefficients (r) of 0.94 for Cu, 0.96 for Zn and 0.95 for Cd, respectively. As expected, the contents of metals extracted by NH_4_Cl were lower than those extracted by HCl, the average percent of extracted Cd was 53%, while for Cu and Zn were 24% and 16%, respectively. Significant positive correlations were found between the pseudo-total metal contents and the metals extracted in NH_4_Cl: Cu (r=0.55) and Cd (r=0.72), while for Zn, the correlation was not significant. Generally, the percent of metals extracted both in HCl and NH_4_Cl were in good agreement with the extractability ranges reported by Kashem et al. [19] for contaminated soils, except for Cu, that in our study was highly extracted by NH_4_Cl.

**Table 2:** The extractable metal contents in diluted acid and in neutral salt solution

**Sites**		**1M HCl (mg kg**^**-1**^**dw)**			**1M NH**_**4**_**Cl (mg kg**^**-1**^**dw)**		
		**Cu**	**Zn**	**Cd**	**Cu**	**Zn**	**Cd**
BM	Mean	40.0	176	0.95	11.7	68.6	0.76
	Range	14.7-73.3	85.0-253	0.34-1.56	7.01-18.0	32.1-124	0.28-1.20
RC	Mean	33.4	117	0.64	9.55	59.5	0.38
	Range	11.8-46.8	52.6-166	0.40-1.06	6.31-17.2	31.0-102	0.21-0.78
SA	Mean	35.5	119	0.36	13.4	64.8	0.24
	Range	13.4-84.2	43.1-145	0.13-0.83	8.03-22.0	29.1-109	0.09-0.55

### Contents of metals in soil solution and measured by DGT

Table 3 shows the metals concentrations in soil solutions (*C*_*soln*_) obtained by centrifugation of the soil slurries used for DGT deployments and metal concentrations in the eluents of the DGT resins (*C*_*DGT*_). The amounts of metals measured as *C*_*soln*_ and *C*_*DGT*_ were considerably lower than those extracted in HCl and NH_4_Cl. In soil solution, concentrations of Zn were found in the range of 31–67 μg L^-1^ (average of 47 μg L^-1^), Cu in the range of 25–66 μg L^-1^ (average of 40 μg L^-1^), while Cd was one order of magnitude lower than Cu and Zn, ranging between 0.24-1.4 μg L^-1^ (average of 0.67 μg L^-1^). For Zn and Cd significant positive correlation was found between C_DGT_ and C_soln_, while Cu *C*_*DGT*_ was weakly related to *C*_*soln*_. The R ratio *C*_*DGT*_*/C*_*soln*_ (0 < R < 1) indicates the capacity of the soil solid phase to resupply the soil solution with metals. When R > 0.95, the metal is present as mobile and kinetically labile species in the solid phase and the capacity of the solid phase to resupply the pore water is high. An R value approaching 0 suggests very limited or no metal resupply from the solid phase [20]. The R ratios for Zn, Cd, and Cu ranged between 0.39–0.74, 0.24–0.84 and 0.20–0.63, respectively, indicating an intermediate case for metal resupply from the solid phase. Also, it seems that Zn and Cd are resupplied more easily than Cu from the soil solid phase.

**Table 3:** **Contents of metals in soil solutions (C**_**soln**_**) and metals measured using DGT technique (C**_**DGT**_**) (Means±SD, n=3)**

**Sites**	**C**_**soln**_**(μg L**^**-1**^**)**			**C**_**DGT**_**(μg L**^**-1**^**)**		
	**Cu**	**Zn**	**Cd**	**Cu**	**Zn**	**Cd**
1	36±2.3	31±0.5	1.0±0.12	13±1.6	15±1.6	0.51±0.08
2	29±2.0	53±0.7	1.4±0.16	13±1.8	36±2.8	0.65±0.07
3	35±3.3	34±0.3	0.98±0.09	8.3±0.9	19±1.5	0.53±0.09
4	51±3.1	47±0.5	0.68±0.11	20±1.4	28±1.4	0.48±0.07
5	28±2.6	55±0.9	0.64±0.15	10±0.8	28±2.1	0.33±0.05
6	53±5.5	58±1.3	0.57±0.07	17±1.3	33±2.4	0.26±0.04
7	44±2.0	64±1.0	0.59±0.17	5.0±0.7	31±2.7	0.44±0.06
8	66±3.2	67±1.1	1.1±0.13	22±1.9	43±2.7	0.48±0.07
9	25±2.6	50±1.1	1.0±0.08	5.0±0.6	29±2.8	0.29±0.10
10	32±3.1	35±0.7	1.3±0.13	6.6±0.7	25±1.9	0.76±0.09
11	50±2.8	61±1.5	0.74±0.06	14±1.2	42±2.5	0.19±0.03
12	46±3.9	58±1.3	0.29±0.04	18±1.5	28±2.3	0.16±0.03
13	28±2.5	46±1.5	0.24±0.05	8.4±0.7	21±1.7	0.19±0.02
14	27±2.6	56±0.8	1.3±0.07	16±1.2	33±1.9	0.46±0.02
15	36±1.8	63±1.6	0.56±0.05	23±2.1	43±2.2	0.15±0.03
16	42±1.9	31±1.0	0.51±0.10	14±1.0	14±1.5	0.12±0.02
17	31±2.2	38±1.6	0.42±0.05	12±1.1	20±1.7	0.33±0.03
18	35±1.5	41±0.7	0.51±0.03	12±0.9	20±1.6	0.36±0.03
19	66±3.6	36±0.9	0.25±0.04	20±1.9	16±1.4	0.08±0.02
20	40±2.0	31±0.2	0.38±0.08	16±1.7	23±1.8	0.12±0.03
21	33±3.2	49±0.4	0.69±0.10	12±0.9	28±1.7	0.24±0.06
22	31±1.6	50±0.4	0.30±0.02	10±0.8	30±2.7	0.17±0.03
23	41±2.9	31±0.6	0.65±0.08	16±1.3	15±1.5	0.18±0.03
24	28±1.3	37±0.8	0.38±0.07	12±1.0	20±1.5	0.28±0.07
25	39±1.5	47±1.0	0.42±0.11	13±1.0	19±1.9	0.14±0.03
26	36±1.8	49±0.9	0.28±0.06	14±1.5	24±1.8	0.19±0.05
27	44±2.6	49±0.6	0.73±0.07	9.3±1.6	22±1.6	0.30±0.05
28	42±2.6	53±1.5	0.99±0.05	10±0.9	21±1.8	0.39±0.04
29	58±2.8	52±1.4	0.76±0.08	17±1.8	34±2.2	0.40±0.05
30	52±1.6	36±1.1	0.29±0.05	20±1.9	23±2.9	0.17±0.03

Note. BM: 1–10; SA: 11–20; RC: 21–30.

Table 4 presents the values of particle concentration (*Pc*) of soils in each sampling point, the *R*_*diff*_ values calculated using 2D DIFS model and the effective concentration (*C*_*E*_) calculated as the ratio between C_DGT_ and *R*_*diff*_. Over-all, homogenous values of *P*_*c*_ were observed for soils collected in gardens from the same village, with the lowest values in SA. The average values of effective concentration, C_E_, decreased in the order: Zn > Cu > Cd, similarly with that observed for soil solution. The measured *C*_*E*_ was, in general, lower but with similar decreasing trend to that reported by Soriano-Disla et al. [12]. Also, *C*_*E*_ values for Cu were comparable with those reported by Ruello et al. [21] for industrial contaminated soils.

**Table 4:** **Particle concentation (P**_**c**_**), R**_**diff **_**calculated by 2D DIFS model and bioavailable metals estimated by determination of effective concentration (C**_**E**_**)**

**Sample sites**	**P**_**c**_**(g cm**^**-3**^**)**	**R**_**diff**_			**C**_**E**_**(μg L**^**-1**^**)**		
		**Cu**	**Zn**	**Cd**	**Cu**	**Zn**	**Cd**
1	4.11	0.0398	0.0407	0.0407	333	361	12.4
2	4.25	0.0395	0.0404	0.0404	318	888	16.1
3	4.00	0.0400	0.0410	0.0409	208	465	12.8
4	4.38	0.0391	0.0400	0.0400	516	689	12.0
5	4.55	0.0388	0.0397	0.0397	258	716	8.37
6	4.36	0.0392	0.0401	0.0401	437	814	6.53
7	4.22	0.0395	0.0404	0.0404	378	765	10.8
8	4.01	0.0400	0.0410	0.0409	547	1050	11.8
9	4.00	0.0400	0.0410	0.0409	126	710	7.10
10	3.56	0.0412	0.0422	0.0422	161	603	18.0
11	3.82	0.0406	0.0415	0.0415	357	1020	4.69
12	3.66	0.0410	0.0419	0.0419	435	680	3.88
13	3.89	0.0403	0.0412	0.0412	208	502	4.50
14	3.16	0.0426	0.0436	0.0435	386	765	10.5
15	4.02	0.0400	0.0410	0.0409	567	1050	3.67
16	4.12	0.0398	0.0407	0.0407	345	348	3.06
17	3.81	0.0406	0.0416	0.0415	292	470	7.90
18	3.55	0.0412	0.0422	0.0422	298	482	8.51
19	3.56	0.0412	0.0422	0.0422	482	391	1.84
20	4.08	0.0399	0.0409	0.0408	412	560	2.86
21	4.23	0.0395	0.0404	0.0404	316	686	5.92
22	4.44	0.0389	0.0399	0.0398	263	765	4.29
23	4.29	0.0393	0.0403	0.0402	412	381	4.49
24	5.06	0.0377	0.0386	0.0385	314	523	7.14
25	4.91	0.0380	0.0389	0.0388	335	498	3.68
26	4.62	0.0387	0.0396	0.0395	371	594	4.69
27	4.29	0.0393	0.0403	0.0402	237	558	7.55
28	4.21	0.0395	0.0404	0.0404	261	524	9.60
29	4.15	0.0396	0.0405	0.0405	422	827	9.82
30	4.52	0.0388	0.0397	0.0397	520	579	4.29

Note. BM: 1–10; SA: 11–20; RC: 21–30.

### Metals concentrations in vegetables

The food chain represents one of the main sources of human exposure to soil contamination through consumption of vegetables. In the Table 5 the concentrations of metals accumulated in the roots of vegetables are shown. Despite the relatively high total metal contents in garden soils, their concentration in roots were similar to metals concentrations typically found in agricultural crops (Cu: 4–15 mg kg^-1^ dw; Zn: 15–200 mg kg^-1^ dw; Cd: 0.2 - 0.8 mg kg^-1^ dw) [22], probably due to the low concentrations in soil pore water and low effective concentrations in soil. The highest metal concentrations were found in carrots, while in garlic and onion the concentrations were comparable. In vegetables from BM area the metals concentrations were higher than in those from RC and SA.

**Table 5:** Metals in roots of vegetables collected from the study area

**Sites**		**Carrot (mg kg**^**-1**^**dw)**			**Onion (mg kg**^**-1**^**dw)**			**Garlic (mg kg**^**-1**^**dw)**		
		**Cu**	**Zn**	**Cd**	**Cu**	**Zn**	**Cd**	**Cu**	**Zn**	**Cd**
BM	Mean	4.22	39.5	0.60	2.75	34.5	0.32	3.07	38.7	0.28
	Range	2.41-7.32	19.5-77.9	0.27-0.92	1.45-3.60	21.7-57.6	0.17-0.59	1.85-4.30	25.6-64.5	0.09-0.46
RC	Mean	3.39	33.8	0.28	2.51	24.9	0.16	2.68	29.0	0.22
	Range	2.10-6.50	21.7-48.6	0.06-0.64	2.05-3.20	17.9-44.1	0.05-0.27	2.10-3.31	17.8-46.5	0.03-0.44
SA	Mean	4.29	32.3	0.12	2.68	27.9	0.17	2.68	33.3	0.14
	Range	2.04-5.70	19.9-64.3	0.04-0.34	1.81-3.51	18.3-48.6	0.05-0.42	1.77-3.55	21.7-53.6	0.03-0.35

The averages of bioavailability factors (*BFs*) calculated as the ratio between the pseudo-total metal content in the plant roots and in soils were Cu 0.086, Zn 0.094 and Cd 0.481 for carrots, Cu 0.058, Zn 0.076 and Cd 0.372 for onion, and Cu 0.063, Zn 0.088 and Cd 0.329 for garlic, respectively. The *BFs* followed the order: Cd > Zn > Cu reported in other studies [23,24]. Also, the *BFs* were of the same order of magnitude with those obtained by Miclean et al. [25] for vegetables collected from Baia Mare area.

### Multivariate statistics

Multivariate statistical approaches are considered very effective for the visualization of relationship between the variables of a multi-dimensional dataset [26], Principal Component Analysis (PCA) and Agglomerative Hierarchical Clustering (AHC) were used to study the specific behaviour of metals in soil plant system, taking into account their total and available contents in soil, accumulation in vegetable, and the soil properties (pH, OC, CEC). The varimax rotated factor loadings of principal components (PCs) are presented in Table 6. Five PC’s with eigenvalues higher than 1 explains 78.2% of the system variance. The first PC exhibits 34.2% of the total variance with positive loadings on pseudo-total metal contents in soil and indicates the common sources of contamination with these metals, represented probably by the mining activities. This latent factor contains also the metals extracted in diluted strong acid showing the good correlations among the pseudo-total and 1M HCl extractable metal contents. The significant positive loading of Cd extracted in 1M NH_4_Cl from this PC can be explained by its high extractability in this solution.

**Table 6:** Varimax rotated factor loadings of experimental variables

**Variable**	**PC1**	**PC2**	**PC3**	**PC4**	**PC5**
Cu AR	**0.878**	0.086	0.189	0.062	0.226
Zn AR	**0.843**	−0.018	0.112	0.303	0.115
Cd AR	**0.879**	0.152	−0.026	0.176	−0.154
Cu HCl	**0.824**	−0.146	0.184	−0.082	0.251
Zn HCl	**0.852**	0.059	0.123	0.298	0.149
Cd HCl	**0.825**	0.239	−0.037	0.235	−0.062
Cu NH_4_Cl	0.308	−0.116	**0.592**	0.090	**0.589**
Zn NH_4_Cl	0.323	−0.117	−0.105	**0.736**	0.003
Cd NH_4_Cl	**0.628**	**0.524**	0.000	0.116	−0.241
Cu soln	0.016	−0.153	**0.728**	0.262	0.235
Zn soln	0.161	−0.022	−0.088	**0.800**	0.250
Cd soln	0.116	**0.749**	−0.037	0.310	−0.152
Cu C_E_	0.212	−0.293	**0.563**	0.146	**0.529**
Zn C_E_	0.257	0.058	0.031	**0.842**	0.160
Cd C_E_	0.290	**0.801**	−0.036	0.126	−0.234
pH	0.353	**−0.732**	0.051	0.228	0.106
OC	−0.114	−0.170	**0.768**	0.145	0.203
CEC	0.256	0.106	**0.834**	−0.088	−0.111
Cu carrot	0.167	0.036	**0.661**	0.258	0.396
Zn carrot	0.028	0.139	0.235	**0.836**	−0.039
Cd carrot	0.081	**0.882**	−0.077	0.027	0.132
Cu onion	0.270	−0.010	0.313	0.348	**0.601**
Zn onion	0.182	0.180	0.468	**0.723**	0.103
Cd onion	0.104	**0.660**	−0.273	0.278	0.275
Cu garlic	0.003	0.203	0.221	0.285	**0.715**
Zn garlic	0.331	0.193	0.293	**0.686**	0.193
Cd garlic	0.143	**0.770**	0.144	−0.033	0.199
Explained variance %	34.2	18.5	11.6	9.4	4.5

Note. The highest loadings are in boldface type.

The second latent factor, responsible for 18.5% of the total variance, shows the correlations between bioavailable Cd and its accumulation in vegetables as suggested by C_soln_, *C*_*E*_, and Cd in roots of all analysed vegetables along with the negative loading of pH. Thus pseudo-total and acid extractable contents were found to be non effective for the prediction of Cd accumulation in plants. Our results are in agreement with other studies [27-29] that reported good correlation between bioavailable Cd in soil measured by DGT and its accumulation in several plant species. However, in other studies this correlation was not observed [12,30]. Although, due to their similar chemistry, it was expected that Cd and Zn would have the same behaviour in soil plant system, but only in case of Cd, the metal content in vegetables were negatively correlated to soil pH. A possible explanation could be the easier soil-plant transfer of Cd by binding to enzymes when the Cd and Zn enter simultaneously in the vegetable cells [24]. Our results were well in line with those obtained by Wang et al. [31] that reported stronger negative correlations between the soil pH and the metal accumulated in plant roots for Cd than for other elements.

The third PC explains about 11.6% of the variability and is determined by the *C*_*soln*_ and *C*_*E*_ of Cu, positively correlated with the OC, CEC and Cu accumulated in carrot roots. Positive, but less correlated appear to be the bioavailable fractions estimated by NH_4_Cl extraction of Cu in soil. The association of Cu in soil solution with soil OC can be explained by the strong complexes formed with dissolved organic carbon [32]. Moreover dissolved organic carbon acts as a major driver of Cu speciation in soil solution and transfer to plants [33] and is presumed that Cu complexes with fulvic and humic acids from soil solution can be easily uptake by some plants [34]. Our results are consistent with those reported by Wang et al. [31] who found positive correlation between soil OC and Cu accumulation in plant roots. In addition, our results confirm the lab-scale studies that found positive correlations between available Cu content assessed by DGT and plants uptake [10,11,35,36], although there are other studies indicated that DGT is less effective in Cu bioavailability prediction [4].

The fourth PC exhibits 9.4% of the total variability, with positive loadings on bioavailable Zn fractions *C*_*soln*_, *C*_*E*_, *C*_*NH4Cl*_, and Zn accumulated in roots of all vegetable species, indicating the correlations between Zn available fractions and their bioaccumulation in the plant roots. Also, the lack of correlation among the pseudo-total and acid extractable Zn contents with Zn accumulation in plants was observed. The strong positive correlations between *C*_*E*_ and plants uptake of Zn observed in our study is confirmed by previous pot experiment studies [4,12,36,37].

The last PC (4.5% variability) is influenced by Cu contents accumulated in roots of onion and garlic that are positively correlated with Cu extracted by NH_4_Cl and Cu *C*_*E*_.

**Figure 1:**
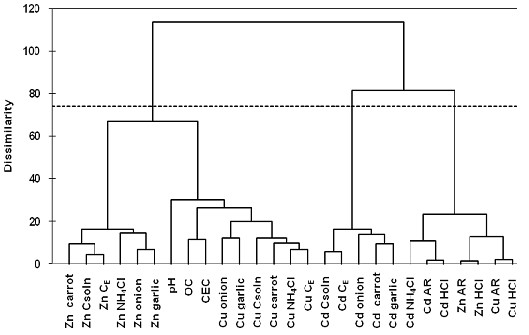
**Dendogram showing the clustering of trace metals and physicochemical soil properties.** Legend. C_E_ – effective concentration; OC – organic carbon; CEC – cation exchange capacity, AR – *aqua regia* (pseudo-total concentration), Csoln – concentration in soil solution.

As presented in Figure 1, the 27 variables were grouped into 3 statistically significant clusters with a dissimilarity of 75%. The cluster C1 is separated in two sub-clusters: the first sub-cluster groups Zn bioavailable concentrations (*C*_*NH4Cl*_, *C*_*soln*_, *C*_*E*_) and Zn accumulated in vegetable roots, while the second sub-cluster contains Cu available concentrations (*C*_*NH4Cl*_, *C*_*soln*_, and *C*_*E*_) and Cu accumulated in vegetable roots along with the soil pH, OC and CEC. Cluster 2 includes Cd in bioavailable forms (*C*_*soln*_, *C*_*E*_) and Cd in vegetable roots suggesting that for the prediction of Cd uptake by vegetables *C*_*soln*_ and *C*_*E*_ are more suitable than chemical extractions. The third cluster contains *aqua regia* and HCl extractable content of the three metals and Cd extracted in NH_4_Cl indicating the strong correlations among these extraction methods, but without clear indication of metals availabilities for plants.

This study is one of the first applications of DGT technique in assessing metals phytoavailability in field conditions, in moderately contaminated soils. The multivariate statistics (PCA and AHC) were used to find the relationships between metals bioavailability in soils assessed using DGT technique and chemical extractions and metals accumulation in plants and to reveal the specific behaviour of metals in soil root system. Even if the relationship between effective concentrations (*C*_*E*_) of metals were not quantitatively related to accumulation in vegetables, the correlations between the two variables were generally stronger than between metals in vegetables and in soil determined by single extraction methods.

## Conclusions

This study represents the first attempt to identify relationships between the availability of Cu, Zn and Cd assessed using DGT technique and several representative extractions procedures (*aqua regia*, diluted strong acid, neutral salt solution, soil solution) and theirs bioaccumulation in food vegetables using multivariate statistical approaches. Usually, to compare the effectiveness of extraction methods for the bioavailability prediction univariate statistics were applied. The new approach can offer supplementary information for the general characterization of the site for risk assessment purposes. Also, since DGT was used generally for the evaluation of metals bioavailability in pot experiments this study represent one of the first application of this technique under field conditions. The results obtained in this study showed that *C*_*E*_, even if do not offer quantitative information on metals transfer from soil to vegetables, is effective for the general evaluation of the risks associated to toxic metals in soils. Metals concentrations assessed by *aqua regia* or by diluted acid (HCl) extractions are not good predictors for metals accumulation in vegetables for the studied metals. Generally, the extraction in NH_4_Cl was more effective for metals bioavailability estimations than acid extractions, except for the prediction of Cd uptake in garlic and onion. Cu transfer to vegetables, especially in carrots, was found to be strongly influenced by soil OC and CEC than Cd or Zn, while pH has a higher influence on Cd transfer to vegetables than on the other studied metals. Despite the relatively high total metal concentrations in soils, their concentrations in soil solution and effective concentrations in soil were considerably lower, thus the metals contents in roots of vegetables were similar to those typically found in agricultural crops. Although future studies on different types of soils and vegetable species or on soil sampling procedures in field conditions are necessary, the effective concentrations can satisfactorily assess metals bioavailability in soils, even in field conditions.

## Methods

### Site description, soil and vegetable sampling

**Figure 2:**
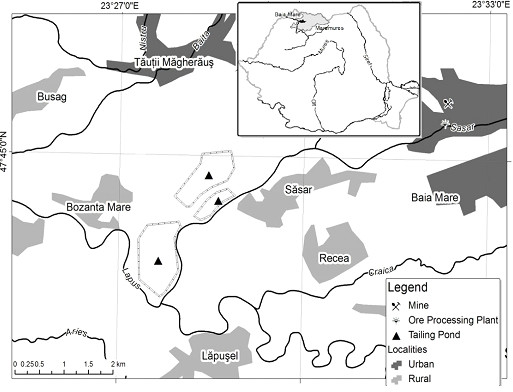
**Schematic map of sampling sites in the Baia Mare mining area.** Legend. The three sampling villages included in the study: Bozanta Mare (BM), Sasar (SA), and Recea (RC).

Soils and vegetables were sampled from private gardens used by locals for growing edible vegetables from three small villages: Bozanta Mare (BM: samples 1–10); Sasar (SA: samples 11–20); and Recea (RC: samples 21–30), in summer of 2011. The villages are located near three tailing deposits resulted from the ore processing activities from Baia Mare, North-West Romania, an area with a long-history of non-ferrous ore mining and processing [18,26]. As presented in Figure 2, the study area is located between 23°24’- 23°30’Est longitude and 47°36’- 47°39’North latitude.

Ten sampling points were randomly selected in each village based on the distribution of the existing gardens. Three common vegetables: Carrot (*Daucus carota*), Onion (*Allium cepa L.*) and Garlic (*Allium sativum L.*) were collected in 7–10 week growth period. In order to assure the comparability of the results, only the roots (considered edible parts) of the plants were used in the assessment study. In each sampling point three specimens of each vegetable species were randomly sampled, then intensely rinsed with tap water and distilled water and stored in polyethylene bags. Once the vegetables removed from the ground, soil samples adhering to their roots and underneath them were sampled. In the lab, the soils were spread over a polyethylene sheet, air-dried at room temperature for one week and sieved through a 2-mm nylon mesh. The vegetable roots were rinsed using 1M HCl, followed by ultrapure water and then dried at 40°C, grounded and sieved through 100-μm nylon mesh. All the soil and vegetable samples were kept in closed plastic bags until analysis.

### Soil and vegetable analysis

The pseudo-total metals concentrations of soils were determined after *aqua regia* digestion according to ISO 11466:1995. An amount of 1 g of dried soil, previously grounded and passed through the 100-μm nylon mesh, was heated with 28 mL *aqua regia*, then filtered through 0.45 μm pore size filter and diluted to 100 mL with ultrapure water. Potentially available contents of metals from soil were determined by single extractions with 1M HCl (ratio w/v = 1:33.3, time 2 h, room temperature) and 1M NH_4_Cl (ratio w/v = 1:6, time 16 h, room temperature), as described by Kashem et al. [19]. For vegetables digestion, an amount of 0.5 g of dried sample was heated with a mixture of 2 mL of H_2_O_2_ and 6 mL HNO_3_ using a MWS3+ Berghoff microwave system (Eningen, Germany), then the resulting solutions were filtered and diluted to 50 mL with ultrapure water. The content of metals were measured by inductively coupled plasma optical emission multichannel spectrometer (ICP-OES) Optima 5300 DV (Perkin Elmer, USA) in soil extracts and by inductively coupled plasma mass spectrometer (ICP-MS), equipped with dynamic reaction cell, ELAN DRC II (Perkin Elmer, Canada) in vegetables, soil solutions and DGT extracts. Soil pH was measured using a JENWAY 3340 pH-meter in 1:5 (w:v) soil to water ratio, while total carbon and inorganic carbon contents were determined by dry combustion and non-dispersive infrared carbon analyzer using the Multi N/C 2100S Analyser (Analytic Jena, Germany). The OC was calculated as difference between total and inorganic carbon. The CEC was calculated after ICP-OES determinations of exchangeable major cations, according to ISO 23470:2007. To measure water holding capacity (WHC), the soil was placed on a filter paper in a vessel containing water until saturation, and then the soil was allowed to drain in water-saturated atmosphere. The water content in saturated soil was determined gravimetrically [28].

Blank samples and certified reference materials (CRMs) of soil (SRM 2709 San Joaquin Soil, New York, USA) and vegetable (IAEA-359 Cabbage, Vienna, Austria) were used for the quality control of total metals determination. Recoveries for all analysed metals from soil CRM were in the range of 87.5–102%, measured with precision between 4.5-12.2% (n=5 parallel samples), while the recoveries for analysed metals in vegetable CRM ranged between 91.0–104% measured with precision ranged between 7.8–13.4% (n=5 parallel samples). Reagents of analytical grade or better and ultrapure water obtained by a Milli Q system (Millipore, France) were used for the experiments.

### DGT and soil solution measurements

DGT devices were purchased from DGT Research Ltd. (Lanchester, UK) and consists of a plastic base covered by a layer of Chelex-100 resin impregnated in a hydrogel to accumulate the metals that passed through the diffusive gel (open pore) layer and protected in exterior by a 0.45-μm filter.

Amounts of 25 g of soil samples were brought to room temperature (22°C), mixed with ultrapure water until 100% WHC, and kept for 24 h at 22°C for equilibration. The DGT devices were gently pushed into the equilibrated soil slurries, ensuring that between the edges of the container and the exposed DGT the distance is at least 2 cm. The containers were covered with plastic films to avoid water evaporation from the soil during the DGT deployment, and kept at 22°C for 24 h. For each soil sample 3 replicates were carried out. At retrieval, the devices were cleaned from the adhering soil by washing carefully with ultrapure water and dried with absorbent paper. Metals were eluted from the chelating resin with 1 mL 1M HNO_3_ for minimum 24 h. The eluents were diluted 5 times before metals determination by ICP-MS.

To determine the mass of a metal accumulated in the resin (M), the equation (1) was used [20]:

(1) $$\;M=CF\;\left(V_{\text{acid}}+V_{\text{gel}}\right)/f_{e}$$

where *C* is the metal concentration in HNO_3_ solution measured by ICP-MS, *F* is the dilution factor (5), *V*_*acid*_ is the volume of HNO_3_ added to the resin (1 mL), *V*_*gel*_ is the volume of resin gel (0.15 mL), f_*e*_ is the elution factor (0.8). The time averaged concentration of metal (*C*_*DGT*_) was calculated by equation 2:

(2) $$\;C_{\text{DGT}}=M\Delta g/\text{D t A}$$

where *Δg* is the thickness of the diffusive gel (0.078 cm) + membrane filter (0.014 cm), *D* is the diffusion coefficient of the metal in the resin gel, *t* is the deployment time (86400 sec), and *A* is the area of the sampling window of the DGT device (3.14 cm^2^).

To measure the metals concentration in soil solution (*C*_*soln*_), a portion of the soil paste prepared for the DGT measurements was introduced in 25 mL polyethylene tubes and centrifuged at 5000 rpm for 20 minutes. The collected supernatant was filtered by means of a syringe connected to a 0.45-μm pore size filter. Soil solutions (3 mL) were stabilized with 10 μL ultrapure 65% HNO_3_ and metals concentrations were measured by ICP-MS. To measure the resupply from solid phase the ratio (*R)* between *C*_*DGT*_ and *C*_*soln*_ was calculated.

The measured DGT flux of metals in the soil can be quantitatively linked to effective concentration (*C*_*E*_) that includes both soil solution concentration and its enrichment from the solid phase, and can be calculated using equation 3:

(3) $$\;C_{E}=C_{\text{DGT}}/R_{\text{diff}}$$

where *R*_*diff*_ is calculated using the computer numerical model 2DDIFS (2DDGT Induced Fluxes in Sediments) as described by Sochaczewski et al. [38]. The required input parameters in order to calculate *R*_*diff*_ were particle concentration (*P*_*c*_), metal diffusion coefficients in water, in soil and in diffusive gels (*D*_*0*_, *D*_*s*_, *D*_*d*_), diffusion layer thickness (*Δg* = 0.092 cm), deployment time (*t* = 24 h). For particle density (*P*_*s*_), the typical value for mineral soils of 2.65 g cm^-3^ was used [39]. A large value for *T*_*c*_ (soil response time) and a small value for *K*_*d*_ (plant available fraction of element bound to the soil) were introduced in the input mode of 2D DIFS according to Tandy et al. [36]. *P*_*c*_ was calculated using equation 4:

(4) $$\;P_{c}=m/V$$

where *m* is the mass of soil used and *V* is the volume of water added to obtain the 100% WHC for DGT deployments.

### Statistical analysis

The XLStat Microsoft Excel plug-in (Addinsoft) was used for the statistical processing of the data. Principal Component Analysis (PCA) with varimax rotation was used to interpret the structure of the main dataset. Agglomerative Hierarchical Clustering (AHC) using the Ward’s linkage method and Euclidian distances as a measure of similarity was used to group the determined parameters into classes.

## Competing interests

The authors declare that they have no competing interests.

## Authors' contributions

MS organized the experimental setting, collected the samples, performed metals determinations, including DGT and chemical extractions, interpreted the results and wrote the manuscript. EAL performed OC analyses and helped at chemical extraction, statistical analysis and manuscript preparation. LRS performed CEC analyses, helped at DGT determinations and manuscript preparation. All authors read and approved the final manuscript.
